# Network Pharmacology-Based Approach to Investigate the Mechanisms of* Hedyotis diffusa* Willd. in the Treatment of Gastric Cancer

**DOI:** 10.1155/2018/7802639

**Published:** 2018-05-02

**Authors:** Xinkui Liu, Jiarui Wu, Dan Zhang, Kaihuan Wang, Xiaojiao Duan, Ziqi Meng, Xiaomeng Zhang

**Affiliations:** Department of Clinical Chinese Pharmacy, School of Chinese Materia Medica, Beijing University of Chinese Medicine, Beijing 100102, China

## Abstract

**Background:**

* Hedyotis diffusa* Willd. (HDW) is one of the renowned herbs often used in the treatment of gastric cancer (GC). However, its curative mechanism has not been fully elucidated.

**Objective:**

To systematically investigate the mechanisms of HDW in GC.

**Methods:**

A network pharmacology approach mainly comprising target prediction, network construction, and module analysis was adopted in this study.

**Results:**

A total of 353 targets of the 32 bioactive compounds in HDW were obtained. The network analysis showed that CA isoenzymes, p53, PIK3CA, CDK2, P27^Kip1^, cyclin D1, cyclin B1, cyclin A2, AKT1, BCL2, MAPK1, and VEGFA were identified as key targets of HDW in the treatment of GC. The functional enrichment analysis indicated that HDW probably produced the therapeutic effects against GC by synergistically regulating many biological pathways, such as nucleotide excision repair, apoptosis, cell cycle, PI3K/AKT/mTOR signaling pathway, VEGF signaling pathway, and Ras signaling pathway.

**Conclusions:**

This study holistically illuminates the fact that the pharmacological mechanisms of HDW in GC might be strongly associated with its synergic modulation of apoptosis, cell cycle, differentiation, proliferation, migration, invasion, and angiogenesis.

## 1. Introduction

Gastric cancer (GC) is one of the most common gastrointestinal malignancies and among the most frequent causes of cancer-related deaths internationally [1]. Although the past decades have seen a decline in morbidity and mortality, as well as a significantly increased 5-year relative survival rate, GC remains a serious public health problem with a dismal survival rate in most regions of the globe [2–5]. Surgical resection is the primary curative therapy for GC, and perioperative chemotherapy and adjuvant chemotherapy as well as adjuvant chemoradiotherapy have been adopted to improve the therapeutic effects of resectable GC [6, 7]. Although the treatment landscape of GC has stridden into the molecular and personalized medicine epoch with the introduction of targeted agents and immunotherapies [8], chemotherapy remains the mainstay of palliative therapy for metastatic GC patients [6]. However, GC usually shows resistance to chemotherapeutics which exhibit a relatively short control of this disease and correlated symptoms, and the survival of most of these patients is less than one year [9, 10]. Furthermore, the application of chemotherapy often produces adverse events such as fatigue, nausea, pancytopenia, and significant gastrointestinal toxicity [11, 12].

As a significant component of complementary and alternative medical systems, traditional Chinese medicine (TCM) has been widely applied to clinically treat cancers for thousands of years in Asian nations, particularly in China and Japan [13]. With mounting clinical practice in cancer comprehensive treatment, TCM has been confirmed to be efficacious not only in alleviating uncomfortable symptoms induced by surgery and chemotherapy, such as fatigue, pain, emesis, diarrhea, and pancytopenia, but also in improving tumor-related symptoms, immune functions, and survival benefits [14–17]. According to a network pharmacology-based study on TCM against stage IV gastric adenocarcinoma, the patients who received TCM treatment exhibited a longer median survival time (18 months) than those without TCM treatment (9 months), with 63.8% 1-year and 17.6% 2-year survival rates [18].* Hedyotis diffusa* Willd. (HDW), an annual herb of the Rubiaceae family, is widely distributed in subtropical area of the world [19, 20]. HDW as a medicinal herb was recorded in Chinese pharmacopoeia (2015 edt), with the functions of inducing diuresis to reduce edema, clearing away the heat evil and detoxification, and promoting blood circulation to arrest pain [20, 21]. Clinically, the herb has often been applied as a critical element in many TCM formulae for the treatment of various cancers, including GC [20, 21]. Meanwhile, the results of a latest retrospective matched-cohort study presented that HDW was the most commonly prescribed single herb for treating GC patients and complementary TCM therapy enhanced the overall survival of patients with GC in Taiwan [22]. Although multiple anticancer activities of HDW have been widely reported [23–27], the molecular mechanisms of HDW against GC remain largely unclear.

**Figure 1:**
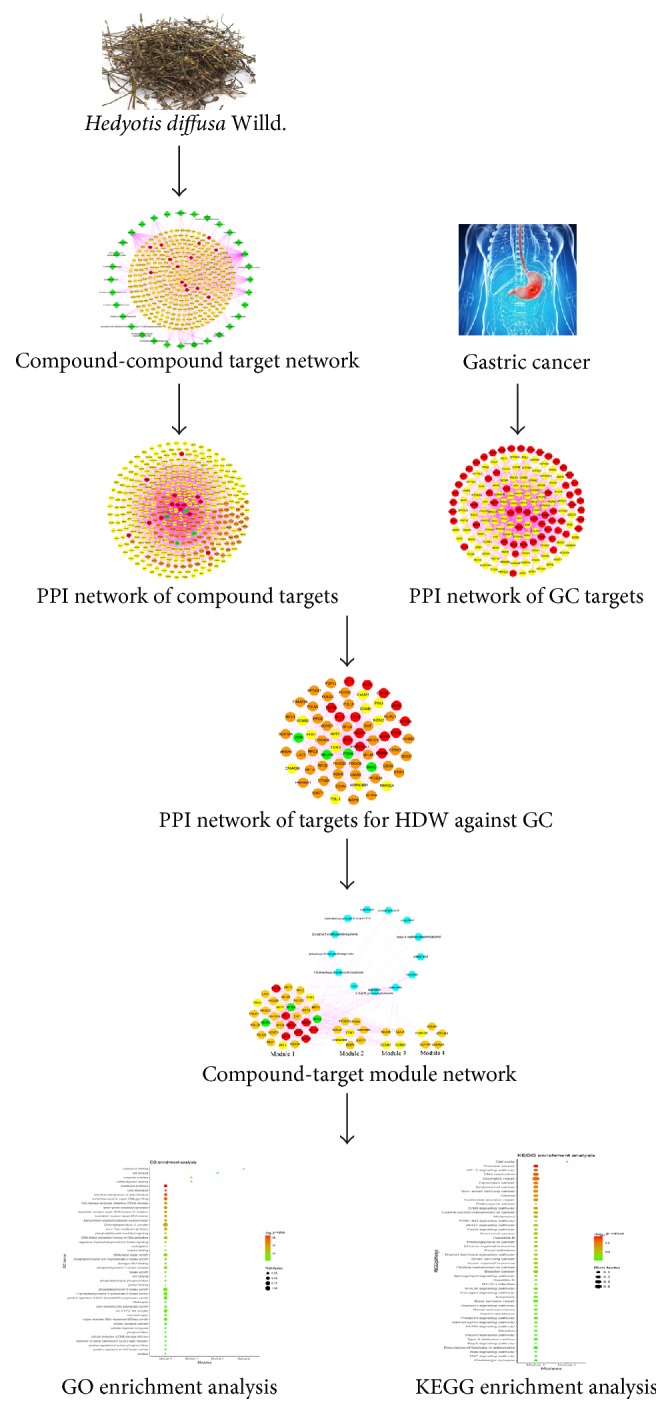
Workflow for HDW in treating GC.

TCM exhibits therapeutic efficacy by the synergistic effects of multicomponent, multitarget, and multipathway, so it is relatively difficult to analyze the intricate mechanisms of TCM merely using traditional experimental approaches [28, 29]. Network pharmacology has emerged as a powerful method incorporating systems biology, bioinformatics, and polypharmacology [30–33], which not only clarifies the complicated interactions among genes, proteins, and metabolites associated with diseases and drugs on a network level, but also coincides with the holistic and systemic views of TCM theory [34]. Thus, we have implemented the network pharmacology approach in an attempt to understand and evaluate the underlying mechanisms of HDW against GC. The workflow of network pharmacology-based study of HDW was shown in Figure 1.

## 2. Materials and Methods

### 2.1. Data Preparation

#### 2.1.1. Herbal Compounds in HDW

To collect the herbal compounds of HDW, we applied Traditional Chinese Medicine Systems Pharmacology Database [35] (TCMSP, http://lsp.nwu.edu.cn/), a unique system pharmacology platform devised for Chinese herbal medicines, and Traditional Chinese Medicine Integrated Database [36] (TCMID, http://www.megabionet.org/tcmid/), which offers a large amount of information regarding formulae and their chemical ingredients. Ninety-three chemical ingredients of HDW were retrieved from the two databases (Table S1), among which some compounds did not have structural information and some were repeated data. Because the targets of the compounds without precise structural information cannot be successfully predicted, these chemicals were removed after deleting the repeated data. Eventually, 42 herbal compounds were gathered (Table S2).

#### 2.1.2. Compound Targets for HDW

The simplified molecular input line entry specification (SMILES) information of all the 42 active compounds was imported into SuperPred [37] (http://prediction.charite.de/), a prediction webserver for Anatomical Therapeutic Chemical (ATC) code and target prediction of compounds. Then 32 herbal chemicals returned their known or predicted targets, and only human targets were reserved. Finally, the information of compound targets was obtained (Table S3).

#### 2.1.3. GC Targets

The human genes associated with GC were acquired from four resources. (1) Therapeutic Target Database [38] (TTD, https://db.idrblab.org/ttd/) is a database to provide information about the known and explored therapeutic protein and nucleic acid targets, the targeted disease, pathway information, and the corresponding drugs directed at each of these targets. We screened TTD with a keyword “gastric cancer” and obtained 12 known GC-related targets. (2) Online Mendelian Inheritance in Man [39] (OMIM, https://omim.org/) is a comprehensive, authoritative, and timely knowledgebase of human genes and genetic disorders compiled to support human genetics research and education and the practice of clinical genetics. We searched OMIM with the keyword “gastric cancer” and collected 12 known GC-related targets. (3) Pharmacogenomics Knowledgebase [40] (PharmGKB, https://www.pharmgkb.org/) is a resource that collects, curates, and disseminates information about the impact of human genetic variation on drug responses. We searched PharmGKB with a keyword “stomach neoplasms” and acquired 37 known GC-related targets. (4) DigSee [41] (http://210.107.182.61/geneSearch/) is a search engine to find explicit association between genes and cancer through biological events. We retrieved DigSee with the keyword “gastric cancer” and in order to make the results more credible we selected 14 known GC-related targets reported by more than or equal to 20 academic papers. After redundancy was deleted, 66 known GC-related targets were eventually collected (Table S4).

#### 2.1.4. Protein-Protein Interaction (PPI) Data

The PPI data came from the Search Tool for the Retrieval of Interacting Genes (STRING) database [42] (https://string-db.org/, ver. 10.5), which provides information regarding the predicted and experimental interactions of proteins. The prediction method of this database comes from neighborhood, gene fusion, cooccurrence, coexpression experiments, databases, and text mining. Furthermore, the database defines PPI with confidence ranges for data scores (low confidence: scores > 0.15; medium > 0.4; high: >0.7). In the present study, PPIs with the combined scores > 0.7 were reserved for further research.

### 2.2. Network Construction

Network construction was performed as follows: (1) Compound-compound target network was established by connecting the herbal compounds and their corresponding targets; (2) PPI network of compound targets was built by connecting the compound targets and other human proteins that interacted with them; (3) PPI network of GC targets was constructed by linking the known GC-related targets and other human proteins that interacted with them; (4) PPI network of targets for HDW against GC was built by intersecting the two networks of (2) and (3).

All the networks were visualized utilizing Cytoscape [43] (http://cytoscape.org/, ver. 3.5.1). The topological features of interaction networks were evaluated by calculating three indices with a Cytoscape tool NetworkAnalyzer, including degree [44], betweenness centrality [45], and closeness centrality [46]. Degree is defined as the number of edges to node i. Betweenness is used for describing the number of shortest paths between pairs of nodes that run through node i. Closeness stands for the inverse of the sum of the distances from node i to other nodes. The higher the three quantitative values of a node are, the greater the importance of the node in the network is.

### 2.3. Module Analysis

The Cytoscape plugin Molecular Complex Detection (MCODE) [47] was applied to analyze clustering modules in the PPI network. In addition, on the basis of the information acquired from Gene Ontology [48] (GO, http://www.geneontology.org) and Kyoto Encyclopedia of Genes and Genomes [49] (KEGG, http://www.genome.jp/kegg/), the GO terms and KEGG pathways enriched by genes in the functional modules were analyzed by employing Database for Annotation, Visualization and Integrated Discovery [50] (DAVID, https://david.ncifcrf.gov/, ver. 6.8) online tool. Meanwhile, the screening principle for significant biological functions and pathways was defined as follows: *p* value < 0.01 and false discovery rate (FDR) < 0.01.

## 3. Results and Discussion

### 3.1. Compound-Compound Target Network

**Figure 2:**
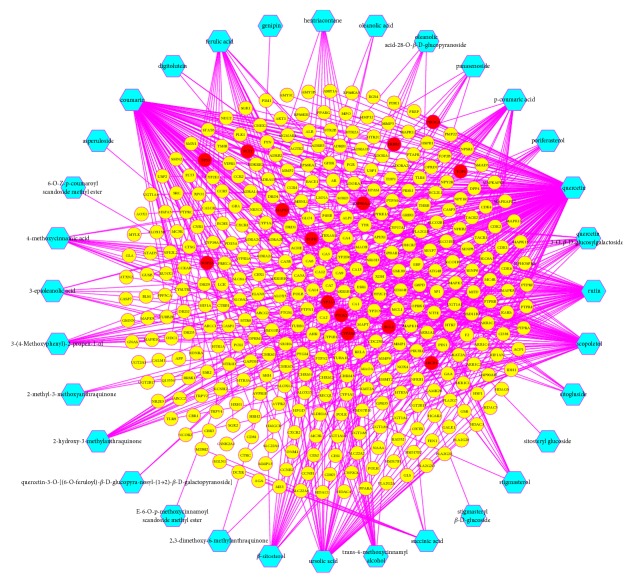
Compound-compound target network (blue hexagons represent compounds of HDW; yellow circles represent compound targets; red circles represent compound/GC targets).

The compound-compound target network consisted of 385 nodes (32 compounds, 339 compound targets, and 14 compound/GC targets) and 733 edges (Figure 2, Table S5). Meanwhile, network analysis showed an average degree value of 22.91 per compound, demonstrating the multitarget treatment characteristics of HDW. Quercetin (degree = 203) and coumarin (degree = 123) exhibited far higher degree values than the other ingredients. Consequently, we contemplated that the top two chemicals probably functioned as critical elements in treating GC. Modern studies have displayed that quercetin exerts powerful antiproliferative and proapoptotic effects on various GC cells by means of multiple mechanisms [51], and quercetin intake is negatively associated with the risk of gastric adenocarcinoma [52]. Moreover, recent evidence implicates that quercetin can also restrain the growth of human GC stem cells [53]. Coumarin is a type of coumarins whose skeletons are characterized by 2H-chromen-2-ones [54]. Coumarins and their derivatives widely found in diverse natural products have become drugs on account of their extensive biological activities like antibacterial, antioxidant, anti-inflammatory, and anticancer effects [54–57]. The present research discovered that it interacted with seven known GC-related targets, including cytochrome P450 2A6 (CYP2A6), cytochrome P450 3A4 (CYP3A4), epidermal growth factor receptor (EGFR), receptor tyrosine-protein kinase erbB-2 (ERBB2), vascular endothelial growth factor receptor 1 (FLT1/VEGFR1), mitogen-activated protein kinase 1 (MAPK1), and prostaglandin G/H synthase 2 (PTGS2), which suggested that coumarin may exert some positive impacts on the treatment of GC. Previous studies have demonstrated that CYP2A6 is an important enzyme responsible for metabolizing coumarin to 7-hydroxycoumarin [58]. Moreover, the latest pharmacological study presents that total coumarins extracted from HDW induce the apoptosis of myelodysplastic syndrome SKM-1 cells by activating caspases and suppressing multiple proteins in phosphatidylinositol 3-kinase (PI3K)/AKT pathway [24]. However, relevant reports investigating the therapeutic efficacy of coumarins and their derivatives on GC and the regulatory effect of coumarin on CYP3A4, EGFR, ERBB2, VEGFR1, and MAPK1 as well as PTGS2 remain deficient nowadays. Therefore, more pharmacological experiments are warranted to validate our computational analysis-based results.

Similarly, many of the potential targets were also connected to multiple herbal ingredients, which reflected the synergistic or additive effects of these ingredients in treating GC. For example, the members of the carbonic anhydrase (CA) family like CA7, CA9, CA12, CA3, CA6, CA14, CA13, CA2, and CA1 were targeted by numerous compounds, which implied that CA isoenzymes probably served as the key targets of HDW. It has long been acknowledged that CAs are extensively expressed in the gastrointestinal tract and play crucial roles in multiple physiological and pathological processes, such as transport of carbon dioxide, pH regulation, ion transport, formation of stomach acidity, bone resorption, calcification, and tumorigenesis [59, 60]. Consider CA9, CA2, and CA1. The expression status of CA9 is closely related to the progression of GC and the regulatory mechanisms of CA9 are relatively complicated [61, 62]. For CA1 and CA2, they are also correlated with gastrointestinal neoplasms and CA2 has been proposed to be a biomarker for gastrointestinal stromal tumors [63]. Furthermore, the compound-compound target network displayed that the chemicals in HDW not only acted on 14 known GC-related proteins but also linked with the other 339 human proteins, which signified that the bioactive compounds of HDW possibly affected diverse targets synergistically and therefore produced potential therapeutic efficacy for other diseases besides GC.

### 3.2. PPI Network of Compound Targets

**Figure 3:**
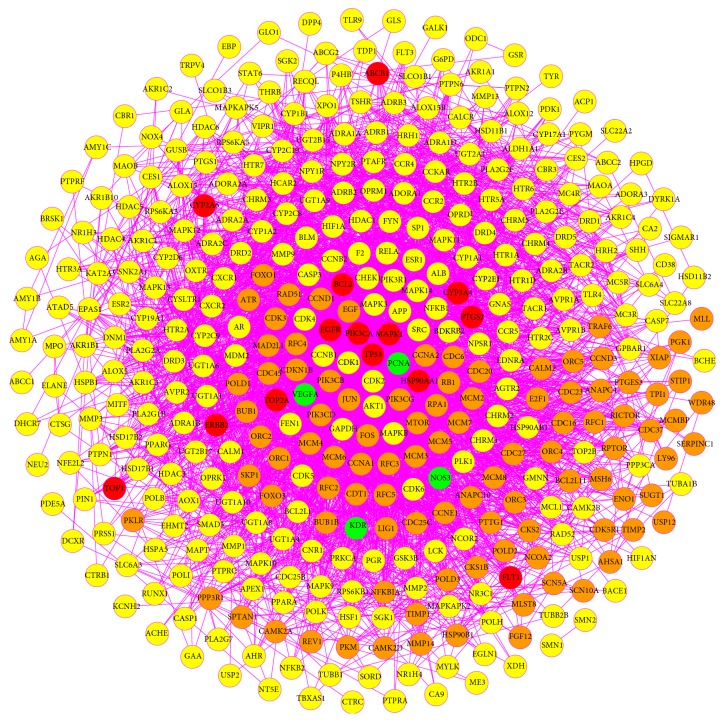
PPI network of compound targets (yellow circles represent compound targets; green circles represent GC targets; red circles represent compound/GC targets; orange circles represent other human proteins that interacted with compound targets or GC targets).

Protein-protein interaction (PPI) networks have been proven to be conducive to decipher the multiple interactions of diverse proteins in some complex diseases including cancer [64, 65]. Thus, the PPI network of compound targets with 409 nodes (293 compound targets, 4 GC targets, 14 compound/GC targets, and 98 other human proteins that interacted with compound targets or GC targets) and 4392 edges (Figure 3, Table S6) was constructed to gain insights into the interactive effects of compound targets modulated by HDW at a system level. In this network, there were 14 intersection targets between compound targets and known GC-related targets, namely, cellular tumor antigen p53 (TP53), phosphatidylinositol 4,5-bisphosphate 3-kinase catalytic subunit alpha isoform (PIK3CA), MAPK1, heat shock protein HSP 90-alpha (HSP90AA1), EGFR, apoptosis regulator Bcl-2 (BCL2), PTGS2, DNA topoisomerase 2-alpha (TOP2A), CYP3A4, ERBB2, CYP2A6, VEGFR1, DNA topoisomerase 1 (TOP1), and multidrug resistance protein 1 (ABCB1). Therefore, the results clearly presented that HDW exerted notable efficacy on GC probably by affecting the whole biological network comprising the 14 targets, in which TP53 (degree = 106) and PIK3CA (degree = 85) should be identified as the pivotal targets in view of their far higher degree values than the other proteins. P53 protein as a tumor suppressor has well established roles in regulating key biological processes like DNA repair, cell cycle arrest, senescence, and apoptosis, and it is altered in more than half of all human cancers, suggesting its significance in preventing cancer [66–68]. About 50% of GC patients have been reported to carry the genetic and epigenetic alterations that lead to the inactivation of p53 [69–71], and* TP53* mutations appear late in precancerous stages of GC bringing about the ultimate transition to cancer [72, 73]. As for* PIK3CA*, it encodes the key enzymatic subunit p110*α* of PI3K [74], and* PIK3CA* functioning as an oncogene plays a key role in GC [75]. Overexpressed* PIK3CA* promoted the invasion and proliferation of GC cells [76], and the upregulation of* PIK3CA* in GC tissues was possibly relevant to lymph node metastasis [76, 77]. Consequently, our findings implied that the components of HDW might produce therapeutic effects on GC by recovering the tumor suppressor activity of p53 and inhibiting the expression level of PIK3CA.

### 3.3. PPI Network of GC Targets

**Figure 4:**
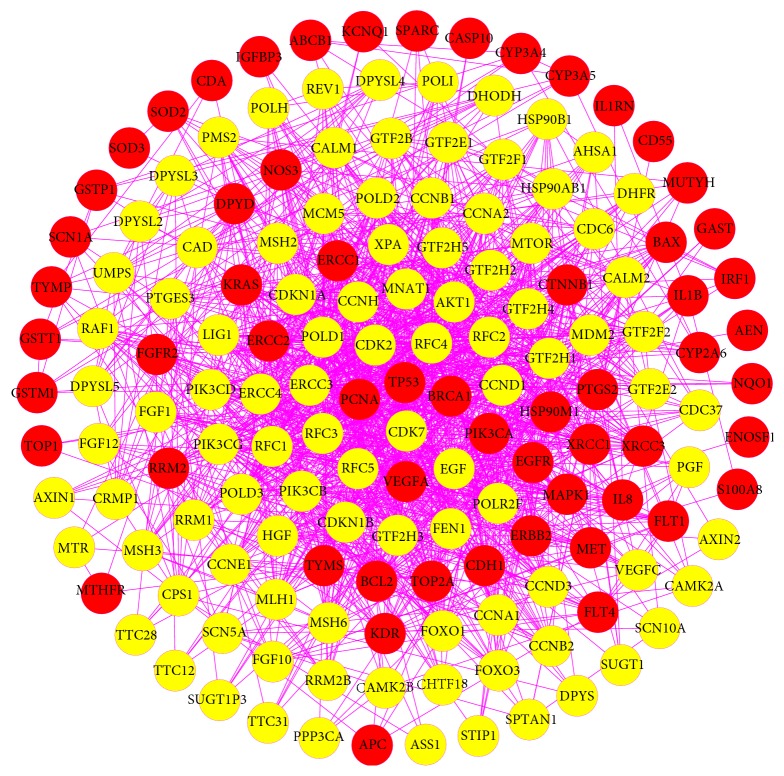
PPI network of GC targets (red circles represent GC targets; yellow circles represent other human proteins that interacted with GC targets).

To discover the relationship between the known GC-related proteins and other human proteins that interacted with them, the PPI network of GC targets was built with 159 nodes (59 GC targets and 100 other human proteins that interacted with GC targets) and 1432 edges (Figure 4, Table S7). Based on the median values for degree, betweenness centrality, and closeness centrality that were 16, 0.00194113, and 0.41253264, respectively, we identified 23 highly connected nodes with degree > 32, betweenness centrality > 0.002, and closeness centrality > 0.413 as significant GC-related targets. Intriguingly, most of the 23 targets were tightly related to cell cycle, such as TP53, cyclin-dependent kinase 7 (CDK7), cyclin-dependent kinase 2 (CDK2), breast cancer type 1 susceptibility protein (BRCA1), G1/S-specific cyclin D1 (CCND1), and CDK-activating kinase assembly factor MAT1 (MNAT1). Obviously, our network analysis revealed that these proteins correlated with cell cycle might play a pivotal role in the tumorigenesis and progression of GC. Consistent with our present study, past findings have confirmed that GC is commonly featured by dysregulated expression of cyclins and other proteins associated with cell cycle [78]. Take CDK2 and cyclin D1 as examples. It is well known that CDKs require binding of cyclins to stimulate cell cycle progression [79, 80], and either continuous proliferation or irregular reentry into cell cycle often occurs due to frequent dysregulation of certain cyclin/CDK complexes caused by tumor-related mutations [81]. Relevant studies have revealed that CDK2 positively modulates the cell cycle of GC, and it can be aberrantly activated by increased malignancy and cancer cell invasion [82]. With regard to cyclin D1, it functions as a critical regulatory factor in the proliferation, apoptosis, invasion, metastasis, and immune escape of tumor cells [83]. And accumulated evidence manifests that overexpressed cyclin D1 is intimately correlated with the progression of GC [84, 85].

### 3.4. PPI Network of Targets for HDW against GC

**Figure 5:**
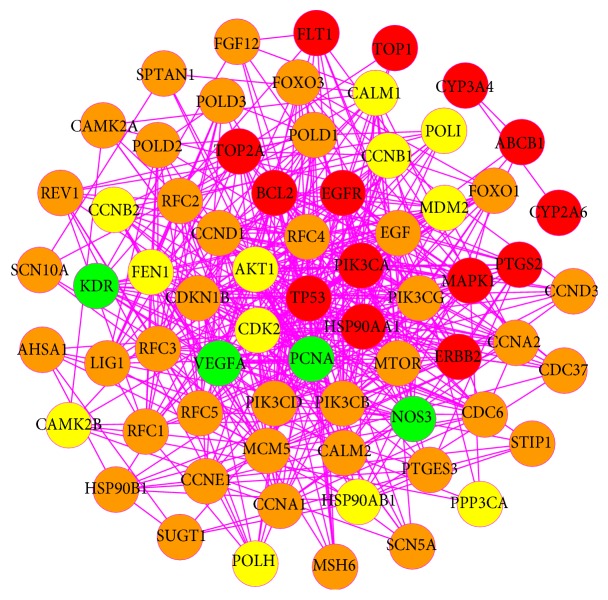
PPI network of targets for HDW against GC (the implication of yellow, green, red, and orange is the same as Figure 3).

In order to further unveil the potential pharmacological mechanisms of HDW against GC, we constructed the PPI network of targets for HDW against GC by intersecting the two networks displayed in Sections 3.2 and 3.3. The network comprised 68 nodes (12 compound targets, 4 GC targets, 14 compound/GC targets, and 38 other human proteins that interacted with compound targets or GC targets) and 474 edges (Figure 5, Table S8). Based on the median values for degree, betweenness centrality, and closeness centrality that were 14, 0.00526562, and 0.463673375, respectively, we identified nodes with the three topological feature values that were higher than the corresponding median values as major targets. Twenty-three targets were reserved after our screening, namely, p53, proliferating cell nuclear antigen (PCNA), CDK2, RAC-alpha serine/threonine-protein kinase (AKT1), PIK3CA, HSP90AA1, replication factor C subunit 4 (RFC4), cyclin-dependent kinase inhibitor 1B (CDKN1B/p27^Kip1^), proepidermal growth factor (EGF), EGFR, BCL2, cyclin D1, vascular endothelial growth factor A (VEGFA), serine/threonine-protein kinase mTOR (MTOR), MAPK1, replication factor C subunit 3 (RFC3), replication factor C subunit 5 (RFC5), G2/mitotic-specific cyclin B1 (CCNB1), replication factor C subunit 2 (RFC2), E3 ubiquitin-protein ligase Mdm2 (MDM2), nitric oxide synthase, endothelial (NOS3), TOP2A, and cyclin A2 (CCNA2).

We could clearly find out that most of these proteins were strongly associated with cell cycle, like CDK2, p27^Kip1^, cyclin D1, cyclin B1, and cyclin A2. Take p27^Kip1^, cyclin B1, and cyclin A2 as examples. P27^Kip1^, an inhibitor of cell cycle, blocks the activation of cyclin E-CDK2 or cyclin D-CDK4 complexes, hence inhibiting G1-S phase transition during the cell cycle progression [86–88]. Meanwhile, P27^Kip1^ is inversely related to GC and its decreased expression is reported as a negative prognostic marker in GC [89–91]. Cyclin B1 regulates the cell cycle transition from G2 to M phase [92] and plays key roles in cell differentiation, apoptosis, and metastasis [93–97]. And its expression might be relevant to the poor outcome of GC patients [98–101]. With regard to cyclin A2, a core regulator of cell division cycle, it binds and activates kinases that regulate S phase and the transition from G2 to M phase [102], and aberrant cyclin A2 expression in human cancers is often correlated with cell proliferation [103, 104]. Furthermore, some proteins were closely related to apoptosis, including TP53, AKT1, BCL2, and MAPK1. And some proteins were intimately relevant to angiogenesis, such as VEGFA and PIK3CA. Consider AKT1, BCL2, MAPK1, and VEGFA. AKT1 is among AKT family members and is detected to be amplified in gastric adenocarcinomas [105, 106]. AKT is a downstream effector of PI3K [107], and the PI3K/AKT signaling pathway participates in apoptosis inhibition and angiogenesis [108]. AKT suppresses apoptosis by inhibiting the actions of BAD and caspase-9 that are related to apoptosis [107]. Moreover, AKT is also intimately relevant to angiogenesis and the invasion of cancer cells into adjacent tissues through VEGF and MMP [109, 110]. As for BCL2, it plays a crucial role not only in promoting cellular survival and inhibiting apoptosis but also in suppressing cellular proliferative activity [111–113]. BCL2 has been reported in GC, and the overexpression of BCL2 serves as an early event in gastric tumorigenesis [114]. Nevertheless, the results from numerous researchers that described the relationship between BCL2 expression and prognosis in GC were sometimes discrepant [115]. With respect to MAPK1, also called extracellular signal-regulated kinase 2 (ERK2), it is a member of the MAP kinase family. The activation of MAPKs regulates diverse cellular processes like proliferation, differentiation, mitosis, and apoptosis [116]. In addition, previous findings showed that miR-197 possibly affected the sensitivity of fluorouracil treatment in a human GC cell line via acting on MAPK1 [117]. As for VEGFA, this protein shows prominent activity in inducing angiogenesis, and VEGFA inhibition has become one of prevalent treatment strategies for multiple cancers [118]. The VEGFA pathway that is critical in promoting tumor angiogenesis serves as a validated target in advanced GC, and the correlation between VEGFA levels and overall survival or stage of GC has been proven [118]. Above all, there is no doubt that cell cycle, apoptosis, and angiogenesis act as vital processes in the development and progression of GC. Accordingly, our present study showed that the simultaneous manipulation of a set of targets associated with cell cycle, apoptosis, and angiogenesis might chiefly account for the curative mechanisms of HDW on GC. In agreement with our study, relevant pharmacological findings suggested that total flavones extracted from HDW could significantly inhibit the proliferation of human GC cells and cause cell cycle arrest in G0/G1 phase, eventually inducing the apoptosis of human GC cells [119]. Moreover, HDW polysaccharides notably induced the apoptosis of human GC cells and exhibited synergy when combined with cisplatin, the mechanisms of which may be correlated with their effects of downregulating BCL2 expression and upregulating p53 expression levels [120].

### 3.5. Module Analysis

**Figure 6:**
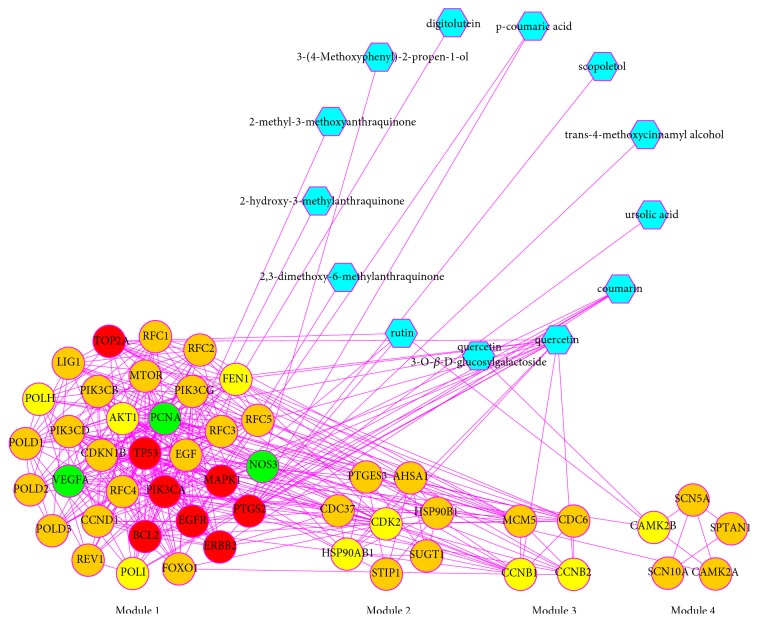
Compound target module network (blue hexagons represent compounds of HDW; the implication of yellow, green, red, and orange is the same as Figure 3; Module 1: MCODE score = 14.312; Module 2: MCODE score = 5.143; Module 3: MCODE score = 4; and Module 4: MCODE score = 3).

**Figure 7:**
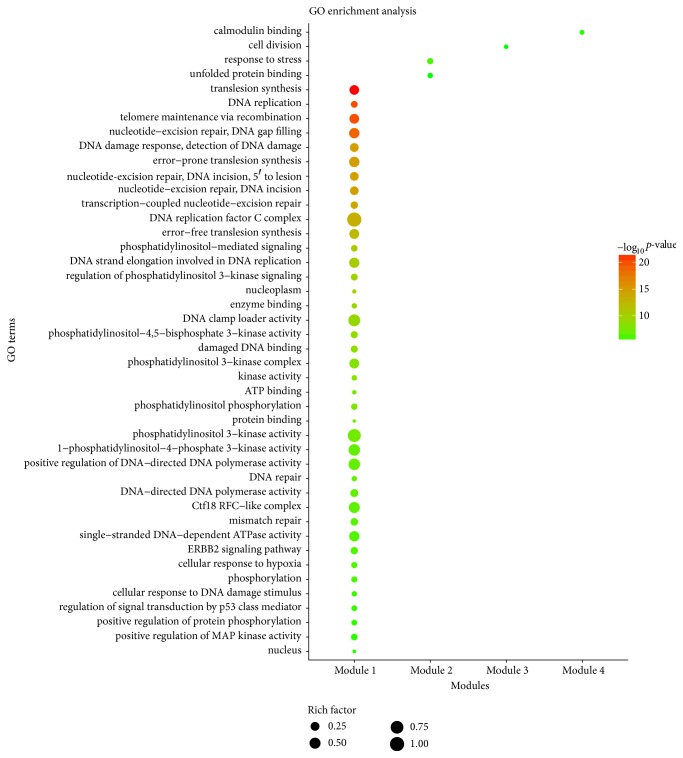
GO enrichment analysis for the targets in different modules (*p* value < 0.01 and FDR < 0.01). The *y*-axis shows significantly enriched GO terms, and the *x*-axis shows different modules. The size of the dot represents the level of rich factor. Rich factor stands for the ratio of the number of target genes belonging to a GO term to the number of all the annotated genes located in the GO term. The higher rich factor represents the higher level of enrichment.

Because clustering modules might represent some pivotal characteristics of PPI networks and probably contain specific biological significance [121], we analyzed the PPI network of targets for HDW against GC by using MCODE and four modules were detected (Figure 6). Meanwhile, we further explored the biological processes, molecular functions, and signaling pathways enriched by the targets in the functional modules in order to clarify the integral regulation of HDW for the treatment of GC. With regard to the GO enrichment analysis, Figure 7 and Table S9 showed the GO terms significantly enriched by the targets in different modules. Module 1 was highly associated with translesion synthesis, nucleotide excision repair, and phosphatidylinositol and its kinase-mediated signaling. Module 2 was highly associated with response to stress and unfolded protein binding. Module 3 was highly associated with cell division. Module 4 was highly associated with calmodulin binding. Thus, we speculated that HDW probably exerted its pharmacological effects on GC by simultaneously involving these biological processes and molecular functions. Nucleotide excision repair (NER), for example, functions as an indispensable and versatile system in maintaining the stability and integrity of the genome, monitoring and repairing multiple DNA damage [122–124]. However, defects in NER would bring about enhanced genomic instability, and unrepaired DNA damage might thereby increase the genetic susceptibility to GC, resulting in the initiation of gastric carcinogenesis [125]. Meanwhile, the disruption of NER system would also alter the chemotherapeutic sensitivity and prognosis of patients with GC [126].

**Figure 8:**
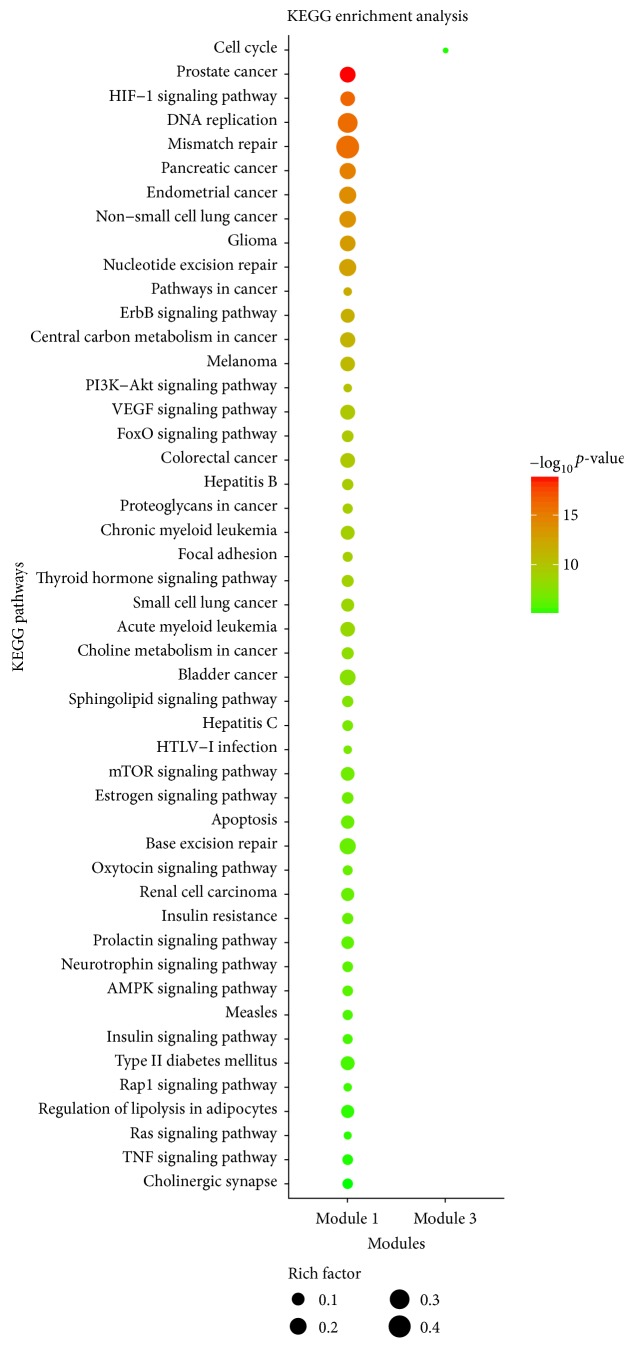
KEGG pathway enrichment analysis for the targets in different modules (*p* value < 0.01 and FDR < 0.01). There were no pathways with FDR < 0.01 in Modules 2 and 4. The *y*-axis shows significantly enriched KEGG pathways, and the *x*-axis shows different modules. The size of the dot represents the level of rich factor. Rich factor stands for the ratio of the number of target genes belonging to a pathway to the number of all the annotated genes located in the pathway. The higher rich factor represents the higher level of enrichment.

**Figure 9:**
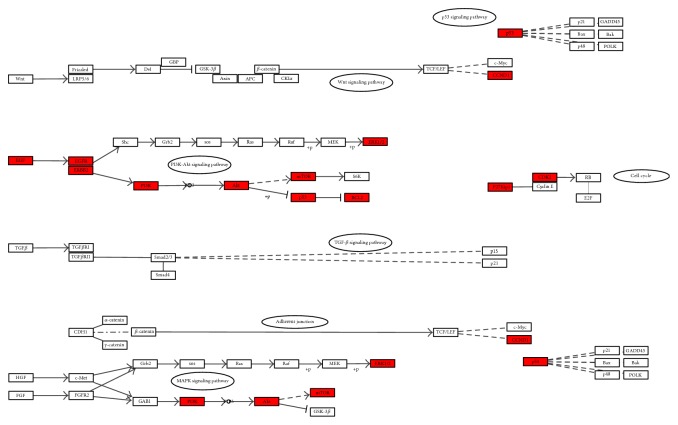
The simplified KEGG pathway of gastric cancer (hsa05226) (the red colored nodes represent the targets in the four modules; the original picture came from KEGG database).

With regard to KEGG enrichment analysis, Figure 8 and Table S10 showed the KEGG pathways significantly enriched by the targets in different modules, and Figure 9 showed the targets of four modules found in the KEGG pathway of gastric cancer (hsa05226). As shown in Figure 8, the targets in different modules were mapped to 48 signaling pathways which can be classified into five categories: human diseases (22/48), environmental information processing (11/48), organismal systems (8/48), genetic information processing (4/48), and cellular processes (3/48). Thus, our findings showed that HDW might integrate diverse signaling pathways to modulate cancers, signal transduction, endocrine system, nervous system, replication and repair, cell growth and death, and cellular community. In addition, we also found that the signaling pathways remarkably enriched by the potential targets of HDW were strongly associated with cell differentiation, proliferation, migration, invasion, apoptosis, cell cycle, and angiogenesis, most of which play a pivotal role in the development and progression of cancers, such as pathways in cancer (hsa05200), PI3K/AKT signaling pathway (hsa04151), VEGF signaling pathway (hsa04370), mammalian target of rapamycin (mTOR) signaling pathway (hsa04150), apoptosis (hsa04210), Ras signaling pathway (hsa04014), and cell cycle (hsa04110). We thereby speculated that the underlying mechanisms of HDW for treating GC may be mainly attributed to its synergistical modulation on the pathways relevant to cancers. As shown in Figure 9, the PI3K/AKT signaling pathway should be identified as the most critical pathway regulated by HDW. The PI3K/AKT signaling pathway is an important growth regulatory pathway that mediates multiple cellular and molecular functions like cell growth, proliferation, metabolism, and survival, as well as angiogenesis, and it is frequently dysregulated in many types of cancers, promoting the tumorigenesis and therapy resistance [127–131]. The PI3K/AKT pathway plays essential roles in the development of GC, and the aberrant activation of this pathway tends to occur in advanced GC patients, with a lower survival rate [131–137]. Previous pharmacological studies confirmed that the chemical compounds of HDW could inhibit the activation of PI3K/AKT pathway by significantly downregulating the expression of PI3K, AKT, and phosphorylated-AKT (p-AKT), inducing the apoptosis of fluorouracil resistant colorectal cancer cells and human myelodysplastic syndrome cells [24, 138]. Blocking angiogenesis has been widely believed to be one of the efficacious treatment choices for inhibiting tumor growth and metastasis considering the essential roles of angiogenesis for tumor growth [139]. However, the long-term use and therapeutic efficacy of angiogenesis inhibitors were largely limited by drug resistance and the cytotoxicity against non-tumor-associated endothelial cells [140]. Fortunately, 4-vinylphenol isolated from HDW has been reported to possess antiangiogenic effects in human endothelial cells, the mechanisms of which may be related to its inhibition on PI3K/AKT, ERK, and p38 signaling pathways, as well as its downregulation on the expression of VEGFR [141]. The other pharmacological study disclosed that HDW extract could inhibit tumor angiogenesis by downregulating the expression of VEGFA in human colon carcinoma cells [140]. It has been universally accepted that the deregulation of cell cycle progression is among the pivotal hallmarks of cancer [142], and the G1/S transition chiefly regulated by cyclin D1 and CDK4 is one of the two main checkpoints that control cell cycle progression [143–145]. Overwhelming evidence has shown that HDW extract could cause cell cycle arrest at the G0/G1 phase, inhibiting the proliferation and inducing the apoptosis of multiple tumor cells, like human colon carcinoma cells, human hepatocellular carcinoma cells, human leukemia cells, and so on [146–148]. To sum up, we speculated that HDW might produce the therapeutic effectiveness on GC by regulating pathways intimately correlated with proliferation, apoptosis, angiogenesis, and cell cycle, in which the PI3K/AKT signaling pathway played more important roles. Nevertheless, we notice that pharmacological findings on HDW for treating GC have been rarely reported. Therefore, more experiments are needed in the future to validate the results of our present study.

## 4. Conclusions

In the present study, we applied a network pharmacology approach to predict, elucidate, and confirm the potential mechanisms of HDW on GC by integrating target prediction, network construction, and module analysis. Firstly, a total of 353 targets affected by 32 bioactive compounds in HDW were obtained, demonstrating a synergistic treatment strategy of TCM featured by multicomponent, multitarget, and multipathway. Secondly, the analysis of compound-compound target network and PPI network of compound targets displayed that quercetin and coumarin probably served as critical constituents in HDW, and CA isoenzymes and p53 as well as PIK3CA might be the key targets of HDW. Thirdly, the analysis of PPI network of GC targets showed that the proteins related to cell cycle regulation may play an essential role in the tumorigenesis and progression of GC. Fourthly, the analysis of PPI network of targets for HDW against GC indicated the therapeutic effectiveness of HDW in GC possibly owing to its simultaneous regulation of the targets relevant to cell cycle, apoptosis, and angiogenesis, such as CDK2, p27^Kip1^, cyclin D1, cyclin B1, cyclin A2, p53, AKT1, BCL2, MAPK1, VEGFA, and PIK3CA. Finally, according to the results of GO and KEGG enrichment analyses, the targets regulated by HDW were significantly correlated with multiple biological pathways, namely, NER, apoptosis, cell cycle, PI3K/AKT/mTOR signaling pathway, VEGF signaling pathway, and Ras signaling pathway, which were involved in the primary pathological processes of GC like apoptosis resistance, dysregulated cell cycle, abnormal differentiation, uncontrolled proliferation, migration, and invasion, as well as angiogenesis.

In summary, the present study provides a systematic method to disclose that the pharmacological mechanisms of HDW on GC might be strongly associated with its synergic modulation on apoptosis, cell cycle, differentiation, proliferation, migration, invasion, and angiogenesis. Moreover, we hope that our study will be beneficial for providing clues to understand and evaluate the synergetic effects of TCM in controlling complex diseases and for facilitating the application of network pharmacology in exploring the potential mechanisms of anticancer TCMs. Nonetheless, further experiments are demanded to validate our findings since this study was performed based on data analysis.

## Abbreviations

HDW: * Hedyotis diffusa* Willd.
GC: Gastric cancer
CA: Carbonic anhydrase
TP53: Cellular tumor antigen p53
PIK3CA: Phosphatidylinositol 4,5-bisphosphate 3-kinase catalytic subunit alpha isoform
CDK2: Cyclin-dependent kinase 2
CDKN1B: Cyclin-dependent kinase inhibitor 1B
CCND1: G1/S-specific cyclin D1
CCNB1: G2/mitotic-specific cyclin B1
CCNA2: Cyclin A2
AKT1: RAC-alpha serine/threonine-protein kinase
BCL2: Apoptosis regulator Bcl-2
MAPK1: Mitogen-activated protein kinase 1
VEGFA: Vascular endothelial growth factor A
PI3K: Phosphatidylinositol 3-kinase
mTOR: Mammalian target of rapamycin.

## Supplementary Materials

Supplementary 1 Table S1: herbal compounds in HDW.

Supplementary 2 Table S2: herbal compounds with structural information.

Supplementary 3 Table S3: herbal compounds in HDW and their corresponding targets.

Supplementary 4 Table S4: known gastric cancer-related targets.

Supplementary 5 Table S5: topological features of all nodes in the compound-compound target network.

Supplementary 6 Table S6: topological features of all nodes in the PPI network of compound target.

Supplementary 7 Table S7: topological features of all nodes in the PPI network of GC targets.

Supplementary 8 Table S8: topological features of all nodes in the PPI network of targets for HDW against GC.

Supplementary 9 Table S9: GO enrichment analysis for the targets in different modules (*p* value < 0.01 and FDR < 0.01).

Supplementary 10 Table S10: KEGG pathway enrichment analysis for the targets in different modules (*p* value < 0.01 and FDR < 0.01).
